# *Anisakis* Contamination in Fillets of Chub Mackerel and Blue Mackerel Sold in Japan

**DOI:** 10.14252/foodsafetyfscj.D-25-00022

**Published:** 2025-09-26

**Authors:** Takahiro Ohnishi, Reiko Teshima, Hiromu Sugiyama

**Affiliations:** 1 Division of Microbiology, National Institute of Health Sciences, 3-25-26 Tonomachi, Kawasaki-ku, Kawasaki-si, Kanagawa 210-9501, Japan; 2 Okayama University of Science, 1-3, Ikoinooka, Imabari-shi, Ehime 794-8555, Japan; 3 Japan Institute for Health Security, National Institute of Infectious Disease, Toyama 1-23-1, Shinjuku-ku,Tokyo 162-8640, Japan

**Keywords:** *Anisakis*, mackerel, contamination

## Abstract

To evaluate the risk of anisakiasis, we investigated their prevalence in fillets of chub mackerel and blue mackerel in 2022 and 2023. The results indicated that the prevalence (percent of larvae-positive fillet samples) and abundance (number of larvae per 100 g fillet) of *Anisakis* larvae in fillets of chub mackerel were not significantly different between the Sea of Japan and the Pacific Ocean. The prevalence and abundance of *Anisakis* larvae in blue mackerel were markedly lower than those in the chub mackerel.

## Introduction

Human anisakiasis is a foodborne illness resulting from the ingestion of raw fish contaminated with *Anisakis* larvae. The number of anisakiasis incidences has rapidly increased, exceeding all other foodborne illnesses, such as *Campylobacter* and *Norovirus* food-borne illnesses, in Japan since 2018^1^^)^. Although *Anisakis* nematodes infect many species of fish, mackerel is an important fish species for human anisakiasis in Japan^1^^)^. Mackerel is generally consumed cooked, vinegared, or as raw slices; however, the most common cause of human anisakiasis in Japan is related to the ingestion of vinegared mackerel^1^^,^^2^^)^.

A previous study suggested that anisakiasis in Japan is primarily caused by *Anisakis simplex* sensu stricto and *A. pegreffii*. *Anisakis simplex* (s.s.) tends to migrate from the viscera to the muscles after death of the fish^3^^)^. In contrast, *A. pegreffii* does not migrate to the muscles as frequently^3^^)^. Therefore, *A. simplex* (s.s.) is mainly associated with human anisakiasis in Japan. A previous report indicated that mackerel caught in the Pacific Ocean were infected with mostly *A. simplex* (s.s.)^3^^)^. In contrast, the contaminant species on fish caught in the Sea of Japan and southern Japan were primarily *A. pegreffiii*^4^^,^^5^^)^.

In this study, we investigated the prevalence of *Anisakis* larvae in fillets of chub mackerel (*Scomber japonicus*) and blue mackerel (*Scomber australasicus*) in 2022 and 2023 to clarify the current situation of *Anisakis* contamination in fish. Investigation of *Anisakis* infections in fish is required to evaluate the risk of human anisakiasis. In this study, we used raw fillets as samples. Fillets are visually inspected, and *Anisakis* larvae found on the surface of the flesh are manually removed. Fatty belly flesh, which contains many *Anisakis* larvae, is often cut from products before shipping. Because these procedures are used for the processing of fish in restaurants and homes, investigations using fillets of fish reflect the risk of actual situations in restaurants and homes more than those using whole fish.

## Materials and Methods

In the present study, we investigated mackerel fillets which were cut away from viscera, head and tail. In 2022 and 2023, we purchased 518 chub mackerel fillets products, including 225 and 293 products fished in the Pacific Ocean and Sea of Japan, respectively. We also purchased 362 blue mackerel fillets products, including 306, 36, and 20 products that were fished in the Pacific Ocean, Southern Japan, and the Sea of Japan, respectively. The lengths from the cranial to caudal end of the chub and blue mackerel fillets were 140 to 400 mm (median: 210 mm) and 170 to 350 mm (median: 210 mm), respectively. These products were purchased randomly from retail stores in Kanagawa Prefecture, Japan. We selected 20 stores before beginning the study and purchased fillet samples in 2022 and 2023. Anisakis larvae on the surface of samples might be manually removed at stores. All the samples were stored at 4 °C and examined within 2 h of purchase. *Anisakis* larvae in whole fillet were detected as described previously^6^^)^. Briefly, the samples were pressed between two glass plates and examined in a light box to visually identify the larvae. After removing the glass plates, the samples were further examined visually under a 4-watt UV lamp operated at a wavelength of 365 nm. Larval DNA was extracted and used as the template for molecular identification^6^^)^.

## Results and Discussion

The prevalence and abundance of *Anisakis* larvae in fillets of mackerel in Japan were investigated in 2022 and 2023. The prevalence of *Anisakis* larvae in all chub mackerel fillet products was 46% (**Table 1**). The abundance was 1.3 and 2.8 larvae/100 g in all and larvae-positive samples, respectively (**Table 1**). The prevalence of *Anisakis* larvae in fillets of chub mackerel caught in the Pacific Ocean was 47%; larval abundance was 1.6 and 3.6 larvae/100 g in all and larvae-positive samples, respectively (**Table 1**). In fillets of chub mackerel caught in the Sea of Japan, *Anisakis* larval prevalence was 46%, with the abundance of 1.2 and 2.5 larvae/100 g in all and larvae-positive samples, respectively (**Table 1**). The prevalence and abundance in the fillets did not differ between chub mackerel caught in the Pacific Ocean and those caught in the Sea of Japan. The obtained isolates from all the chub mackerel comprised 1959 *A. simplex* (s.s.) isolates, one of *A. pegreffii*, and seven of hybrid genotype larvae of *A. simplex* (s.s.) and *A. pegreffii*. Previous studies reported that the dominant *Anisakis* species in chub mackerel in the Sea of Japan is *A. pegreffii*^4^^,^^5^^)^. Because *A. pegreffii* does not migrate to the muscles as frequently, it has been supposed that chub mackerel in the Sea of Japan is relatively safer than that caught in Pacific Ocean. In this study, 1294 *larvae* were isolated from fillets of chub mackerel caught in the Sea of Japan and comprised almost *A. simplex* (s.s.). Because the testing and sampling methods in this study differed from those in previous studies, the results may not be simply compared among the studies. However, it was possible that results in this study suggest the tendency to increase the prevalence of *A. simplex* (s.s.) in chub mackerel caught in the Sea of Japan. In 2018, the incidence of human anisakiasis caused by bonito ingestion dramatically increased in Japan, with a total of 94 incidents^1^^)^. A rise in sea temperature is speculated to have resulted in a change in the migration route of bonito^7^^)^. Changes in the migration route may lead to changes in infection opportunities. The migration route of mackerel could also be affected by climate change.

**Table 1.** Prevalence and abundance of *Anisakis* larvae in chub mackerel products

Region	No. of samples	No. of positive samples	Prevalence (%)	Total weight of all samples (g)	Total weight of positive samples (g)	No. of larvae	Abundance in all samples	Abundance in positive samples
Sea of Japan	293	134	46	104712	51553	1294	1.2	2.5
Pacific Ocean	225	106	47	42145	18900	673	1.6	3.6
Total	518	240	46	146857	70453	1967	1.3	2.8

The prevalence of *Anisakis* larvae in all blue mackerel fillet samples was 5% and abundance was 0.1 and 1.7 larvae/100 g in all and larvae-positive samples, respectively (Table 2). The blue mackerel of which fillets contained Anisakis larvae were caught in the Pacific Ocean (Table 2). The obtained isolates from blue mackerel fillets comprised 53 *A. simplex* (s.s.) isolates. The prevalence and abundance of *Anisakis* larvae in blue mackerel were markedly lower than those in the chub mackerel in this study. The cause of the lower contamination in blue mackerel fillets has not yet been clarified.

**Table 2.** Prevalence and abundance of *Anisakis* larvae in blue mackerel products

Region	No. of samples	No. of positive samples	Prevalence (%)	Total weight of all samples (g)	Total weight of positive samples (g)	No. of larvae	Abundance in all samples	Abundance in positive samples
Pacific Ocean	306	18	6	56660	3209	53	0.1	1.7
Sea of Japan	20	0	0	4170	0	0	0.0	0.0
Southern Japan region	36	0	0	7640	0	0	0.0	0.0
Total	362	18	5	68470	3209	53	0.1	1.7

## References

[1] Ministry of Health, Labour and Welfare, Japan. Annual statistics of food poisoning 2024 [In Japanese]. https://www.mhlw.go.jp/stf/seisakunitsuite/bunya/kenkou_iryou/shokuhin/syokuchu/04.html. Accessed on November 19, 2024.

[2] Suzuki J, Murata R, Kodo Y. Current status of Anisakiasis and *Anisakis* larvae in Tokyo, Japan. Food Safety. 2021; 9(4): 89–100. . 10.14252/foodsafetyfscj.D-21-00004 35004097 PMC8691968

[3] Suzuki J, Murata R, Hosaka M, Araki J. Risk factors for human *Anisakis* infection and association between the geographic origins of *Scomber japonicus* and anisakid nematodes. Int J Food Microbiol. 2010; 137(1): 88–93. . 10.1016/j.ijfoodmicro.2009.10.001 19892425

[4] Quiazon KMA, Yoshinaga T, Ogawa K. Distribution of *Anisakis* species larvae from fishes of the Japanese waters. Parasitol Int. 2011; 60(2): 223–226. . 10.1016/j.parint.2011.03.002 21397715

[5] Umehara A, Kawakami Y, Matsui T, Araki J, Uchida A. Molecular identification of *Anisakis simplex* sensu stricto and *Anisakis pegreffii* (Nematoda: Anisakidae) from fish and cetacean in Japanese waters. Parasitol Int. 2006; 55(4): 267–271. . 10.1016/j.parint.2006.07.001 16942906

[6] Ohnishi T, Banzai A, Hara-Kudo Y, Sugiyama H. Prevalence and abundance of *Anisakis* larvae in ready-to-eat mackerel products in Japan. Int J Food Microbiol. 2023; 395: 110181. . 10.1016/j.ijfoodmicro.2023.110181 37001481

[7] Takano T, Sata N, Iwaki T, et al. Anisakid larvae in the skipjack tuna *Katsuwonus pelamis* captured in Japanese waters: Two-year monitoring of infection levels after the outbreak of human anisakiasis in 2018. Parasitol Int. 2024; 103: 102938. . 10.1016/j.parint.2024.102938 39067843

